# Predictive Modeling of Mental Illness Onset Using Wearable Devices and Medical Examination Data: Machine Learning Approach

**DOI:** 10.3389/fdgth.2022.861808

**Published:** 2022-04-14

**Authors:** Tomoki Saito, Hikaru Suzuki, Akifumi Kishi

**Affiliations:** ^1^JMDC Inc., Tokyo, Japan; ^2^Graduate School of Education, The University of Tokyo, Tokyo, Japan

**Keywords:** machine learning, medical examination, mental illness, mHealth, physical activity, predictive detection, sleep, wearable data

## Abstract

The prevention and treatment of mental illness is a serious social issue. Prediction and intervention, however, have been difficult because of lack of objective biomarkers for mental illness. The objective of this study was to use biometric data acquired from wearable devices as well as medical examination data to build a predictive model that can contribute to the prevention of the onset of mental illness. This was an observational study of 4,612 subjects from the health database of society-managed health insurance in Japan provided by JMDC Inc. The inputs to the predictive model were 3-months of continuous wearable data and medical examinations within and near that period; the output was the presence or absence of mental illness over the following month, as defined by insurance claims data. The features relating to the wearable data were sleep, activity, and resting heart rate, measured by a consumer-grade wearable device (specifically, Fitbit). The predictive model was built using the XGBoost algorithm and presented an area-under-the-receiver-operating-characteristic curve of 0.712 (*SD* = 0.02, a repeated stratified group 10-fold cross validation). The top-ranking feature importance measure was wearable data, and its importance was higher than the blood-test values from medical examinations. Detailed verification of the model showed that predictions were made based on disrupted sleep rhythms, mild physical activity duration, alcohol use, and medical examination data on disrupted eating habits as risk factors. In summary, the predictive model showed useful accuracy for grouping the risk of mental illness onset, suggesting the potential of predictive detection, and preventive intervention using wearable devices. Sleep abnormalities in particular were detected as wearable data 3 months prior to mental illness onset, and the possibility of early intervention targeting the stabilization of sleep as an effective measure for mental illness onset was shown.

## Introduction

Mental illness, including depression, is highly prevalent worldwide, and the lifetime prevalence has been reported to be as high as ~50 and 20% in the United States and Japan, respectively (1, 2). Because mental illness causes a significant decrease in quality of life and socioeconomic loss, its treatment and prevention are among the most serious societal challenges of today (3). Prevention and detection at the onset of mental illness are extremely important because of the generally low remission rate for mental illness and the favorable prognosis of early initiation of treatment (4, 5). Predictive detection is difficult, however, because there are no objective biomarkers for mental illness.

Meanwhile, the spread of wearable sensor technologies has enabled the continuous acquisition of detailed daily lifestyle data (e.g., sleep and activity) from individuals (6). Furthermore, advances in computational processing capabilities and machine learning have enabled the development of predictive models that utilize high-frequency and high-dimensional data linked from wearable devices. Therefore, research combining big health data collection, machine learning, and artificial intelligence (AI) analysis in the health care field has attracted attention, and there are high expectations for their application to the objective examination and onset prediction of mental illness (7–12).

Previous studies have primarily examined the usefulness of sleep and physical activity data. Fang et al. reported that irregular sleep rhythms were associated with worsening of mood and depressive symptoms the following day, indicating the importance of maintaining regular sleep habits for the maintenance and improvement of mental health (13). Nakamura et al. reported that time-series analysis of physical activity data could distinguish depression and schizophrenia (14, 15) and that the depressive scores of healthy and depressed subjects could be continuously estimated from local time-series statistics of physical activity (16). Cho et al. showed the possibility of constructing a predictive model of mood level among patients with mood disorders by applying machine learning to wearable data, such as sleep, activity, and heart rate (17). Sano et al. showed that the application of machine learning to physiological and behavioral data acquired by wearable sensors and mobile phones could be used to classify students' self-reported mental health statuses (18). However, the sample sizes or observation periods of these studies were insufficient, and they have not reached the point where they look beyond symptoms and pathological evaluations to detect disease onset. In particular, the construction of a highly accurate disease onset predictive model that applies machine learning to large-scale data has not yet been achieved.

In addition to wearable data, blood- and urine-sample data collected during regular medical examinations can also be used as biomarker candidates for mental illness. Multiple blood metabolites have been shown to be associated with depression severity, with tryptophan concentration being particularly useful in classifying depressed individuals (19, 20). However, the construction of a highly accurate mental illness onset event predictive model that combines daily behavioral data relating to sleep or physical activities acquired by wearable devices with biochemical data based on blood and urine samples regularly acquired during medical examinations has not been achieved. This is primarily because big data that link medical examination and wearable data did not exist at the time.

The objective of this study was to build a model that can objectively predict the possibility of onset of mental illness from wearable and medical examination data. We have been engaged in the construction of a database that links insurance claims and medical examination data from society-managed health insurance (21), and wearable data linked to personal health records (PHRs), provided by information and communications technology (ICT) services for society-managed health insurance members. The data specifically include sleep, physical activity, and heart rate metrics measured using a consumer-grade wearable Fitbit device. In this study, we built a machine learning predictive model for mental illness onset that was trained using past insurance claims data. This study is meaningful in that the construction of a predictive model of disease onset based on the objective monitoring of human daily life and health status can facilitate the detection and prevention of the onset of mental illness and contribute to resolving widespread mental health issues in society.

## Methods

### Study Design and Dataset

We conducted analyses of a database constructed by JMDC Inc. (21), which includes information on more than seven million society-managed health insurance members in Japan (insured individuals and dependents). This was comprised of our subject base, which is on large-scale and includes insurance claims (e.g., outpatient, hospitalization, and dispensary) and medical examination results. Notably, it also includes a trove of wearable data.

The subjects in this study were chosen from those satisfying the following conditions:

- Society-managed health insurance member;
- Individual whose wearable data are linked to PHRs [service name: Pep Up (https://pepup.life)], which is an ICT service by JMDC Inc. for society-managed health insurance members;
- Individual having a history of continuously linking wearable data for more than 3 months (15+ days per month); and
- Individual undergoing regular medical examinations.

The survey period ran from August 2016 to January 2020. The data were anonymized. Written informed consent was waived owing to the retrospective nature of this study while the subjects agreed to have their data analyzed when linking their data to PHRs. The ethical review of this study was performed by the Ethics Committee of the Research Institute of Healthcare Data Science (RIHDS). Date of approval: October 26, 2020; Approval number: RI2020013. The procedures were carried out in accordance with the approved guidelines.

### Outcome Definition

Mental illness differs by individual in its duration from onset to hospitalization. However, as taken from the insurance claim data, the start of the onset was unclear. Hence, we based the onset period on the time of the first mental illness consultation. Relevant insurance claim information included the following qualifying criteria:

- Administration of hypnotics, anxiolytics, or antidepressants: prescription of medicine corresponding to Anatomical Therapeutic Chemical (ATC) Classification System codes (i.e., N05B, N05C, and N06A) as defined by the European Pharmaceutical Market Research Association (EphMRA);
- Psychiatric visits: calculation of medical practices corresponding to the Quick Reference Table of Medical Score classification codes I002 and I002-2.

Because hypnotics and anxiolytics are effective treatments for mental illness, they are frequently prescribed as a temporarily treatment for surgical hospitalization. Thus, to strictly determine that the administration was connected to a mental illness, we chose the initiation of both “administration of hypnotics, anxiolytics, or antidepressants” and “psychiatric visits” as the qualifying outcome. Henceforth, this combined outcome is referred to as “mental illness onset”. Although this study did not target a specific type of mental illness, this definition approximately narrows the range of the targets to people with depression or anxiety symptoms.

### Exclusion Criteria

Potential subjects who met the following conditions were excluded from the subject data of this study:

- Individuals with mental illness onset prior to linking wearable data, during discontinued linking of data, or within 3 months after starting data linking; and
- Individuals with different months of meeting the qualifying outcomes listed above.

### Wearable Data

The wearable data used in this study were acquired from Fitbit wearable devices from Fitbit Inc. The device model differed among subjects, but high inter-device reliability was reported (22). The data were systematically acquired through the Fitbit application programming interface (API) and consolidated into a single-format database.

The information for sleep-related items collected for each sleep session included *isMainSleep, timeInBed, minutesAsleep, minutesToFallAsleep, minutesAfterWakeup, deepMinutes, remMinutes, lightMinutes, wakeMinutes*, and *startTime* data items. *isMainSleep* is a Boolean that determines whether the given sleep event is main or not, e.g., long- or short-term sleep. *timeInBed* is the time spent in bed; *minutesAsleep* is the total sleep time excluding awakening time; *minutesToFallAsleep* is the time until falling asleep; *minutesAfterWakeup* is the time until getting out of bed after waking up; *remMinutes, ligthMinutes, deepMinutes*, and *wakeMinutes* are the times spent in each sleep stage [i.e., rapid eye movement (REM), light non-REM (NREM), deep NREM, and wakefulness, respectively]; and *startTime* is the bedtime.

The information for activity-related items collected for each day included steps, *veryActiveMinutes, fairlyActiveMinutes*, and *lightlyActiveMinutes*. The physical activity times according to activity intensity were assigned based on the heart rate measured by the Fitbit. The heart rate information was acquired each day from *RestingHeartRate*.

### Medical Examination Data

The medical examination data used in this study were acquired from annual medical examinations, which are required by law to be carried out by businesses affiliated with society-managed health insurers. The measurement items included body mass index (BMI), blood pressure (systolic and diastolic), blood-test items [i.e., triglyceride (TG), high-density lipoprotein (HDL) cholesterol, low-density lipoprotein (LDL) cholesterol, aspartate aminotransferase (AST), alanine aminotransferase (ALT), gamma glutamyl transferase (GT), fasting blood sugar (FBS), and hemoglobin A1c (HbA1c)], urine-test items (i.e., urinary sugar and uric protein qualitative), and interview items related to smoking, drinking, and exercise habits.

### Data Preprocessing

The predictive model in this study predicted the presence of the onset of mental illness within 1 month using three continuous months of Fitbit and medical examination data closest to (i.e., within or prior to) the period. When a single subject had multiple time series that could be used as training data, all of them were used. Only the time series at the time of mental illness onset, however, was used for individuals who actually had mental illness onset.

The features used to build the predictive model are shown in Table 1. The wearable data were collected each month, with monthly features for 3 months being created. These features were labeled “[featurename]n,” where *n* = 1, 2, or 3 for the nth continuous month of data. The sleep data automatically acquired from the Fitbit were used to calculate social jet lag, sleep regularity index (SRI), and chronotype, which express sleep habits that are closely associated with mental health. Social jet lag is an index that indicates circadian misalignment and is calculated as the absolute difference between the mid-point of the sleep hours on weekdays and weekends (23). A larger social jet lag is known to exacerbate various health risks (24, 25). SRI quantifies the regularity of daily sleep–wake rhythms and is calculated as the concordance rate of sleep–wake occurrences in 24-h units (26, 27). Decreased SRI has been associated with stress, depression, poorer academic performance, and increased illness risk. Chronotype indicates morningness–eveningness preference and is often measured using questionnaires (28, 29). It is also associated with mental illness (e.g., depression) (30, 31). In this study, the chronotype calculated from wearable devices was used as an index to calculate corrected mid-sleep time on weekends for those with sleep debt corrected by its magnitude (32).

**Table 1:** Features used in building the predictive model.

**Feature name**	**Unit**	**Description**
**Wearable data [heart rate]**		
RHR	bpm	monthly average of daily RestingHeartRate
**Wearable data [activity]**		
veryActiveMinutes	min	monthly average of daily veryActiveMinutes
fairlyActiveMinutes	min	monthly average of daily fairlyActiveMinutes
lightlyActiveMinutes	min	monthly average of daily lightlyActiveMinutes
Steps	steps	monthly average of daily steps measured
log_count.active	days	monthly number of days for activity data linkage
**Wearable data [sleep]**		
notMainSleep.minutes	min	monthly average of daily minutes of sleep determined to be “isMainSleep=False”
notMainSleep.counts	times	monthly counts of sleep determined to be “isMainSleep=False”
log_count.sleep	days	monthly number of days for activity data linkage
timeInBed	min	monthly average of daily timeInBed
minutesAsleep	min	monthly average of daily minutesAsleep
minutesToFallAsleep	min	monthly average of daily minutesToFallAsleep
minutesAfterWakeup	min	monthly average of daily minutesAfterWakeup
deepMinutes	min	monthly average of daily deepMinutes
remMinutes	min	monthly average of daily remMinutes
lightMinutes	min	monthly average of daily lightMinutes
wakeMinutes	min	monthly average of daily wakeMinutes
startTime.sleep	[hhmmss]	monthly average of daily bedtime
SC_lag	min	monthly social jetlag
chronotype	[hhmmss]	monthly chronotype
SRI	-	monthly Sleep Regularity Index
**Health Examination Data**		
BMI	kg/m^2^	Body Mass Index
SBP	mmHg	Systolic Blood Pressure
DBP	mmHg	Diastolic Blood Pressure
TG	mg/dl	Triglyceride
HDL	mg/dl	HDL cholesterol
LDL	mg/dl	LDL cholesterol
AST	U/l	Aspartate aminotransferase
ALT	U/l	Alanine aminotransferase
GT	U/l	Gamma glutamyl transferase
FBS	mg/dl	Fasting blood sugar
HBA1C	% (NGSP)	HbA1c
US	-	Urinary sugar (1=-, 2 = +/-, 3 = +, 4 = ++, 5 = +++)
UP	-	Uric protein qualitative (1=-, 2 = +/-, 3 = +, 4 = ++, 5 = +++)
**Health examination data [questionnaire]**		
SMOKE	-	Do you habitually smoke? You habitually smoke if you have ever smoked over 100 cigarettes in total or for 6 months or more and have also smoked in the last month. (1 = Yes, 2 = No)
DRINK	-	How often do you drink (sake, distilled spirit, beer, liquor)? (1 = Every day, 2 = Sometimes, 3 = Rarely)
AMOUNT_DRINK	-	Amount of drinking per day on days when you drink. 1 go of sake is equivalent to the following: a medium bottle of beer (around 500 ml), distilled spirit (80 ml), a glass of double whiskey (60 ml), two glasses of wine (240 ml) (1 = <1 go, 2 = 1 go to <2 go, 3 = 2 go to <3 go, 4 = 3 go or more)
FITNESS	-	Do you exercise more than 30 min, more than twice in a week and continue this exercise habit more than 1 year? (1 = Yes, 2 = No)
WALK	-	Do you walk or perform same level of physical activity as walk more than 1 h per day? (1 = Yes, 2 = No)
**Other data**		
GENDER	-	Gender (1 = Male, 2 = Female)
AGE	years	Age at the end of the 3 months
YM	[yyyymm]	the end period of 3 months
YEAR	-	the Year of YM
MONTH	-	the Month of YM

The monthly features of wearable data are described as “[featurename]1–3,” corresponding to the 1st−3rd month of the continuous 3-month data-linked period [e.g., SRI 1 is the SRI of the first month (earliest) of the data-linked period, and SRI 3 is the SRI of the last month (latest) of the data-linked period].

### Machine Learning and Validation

**Figure 1:**
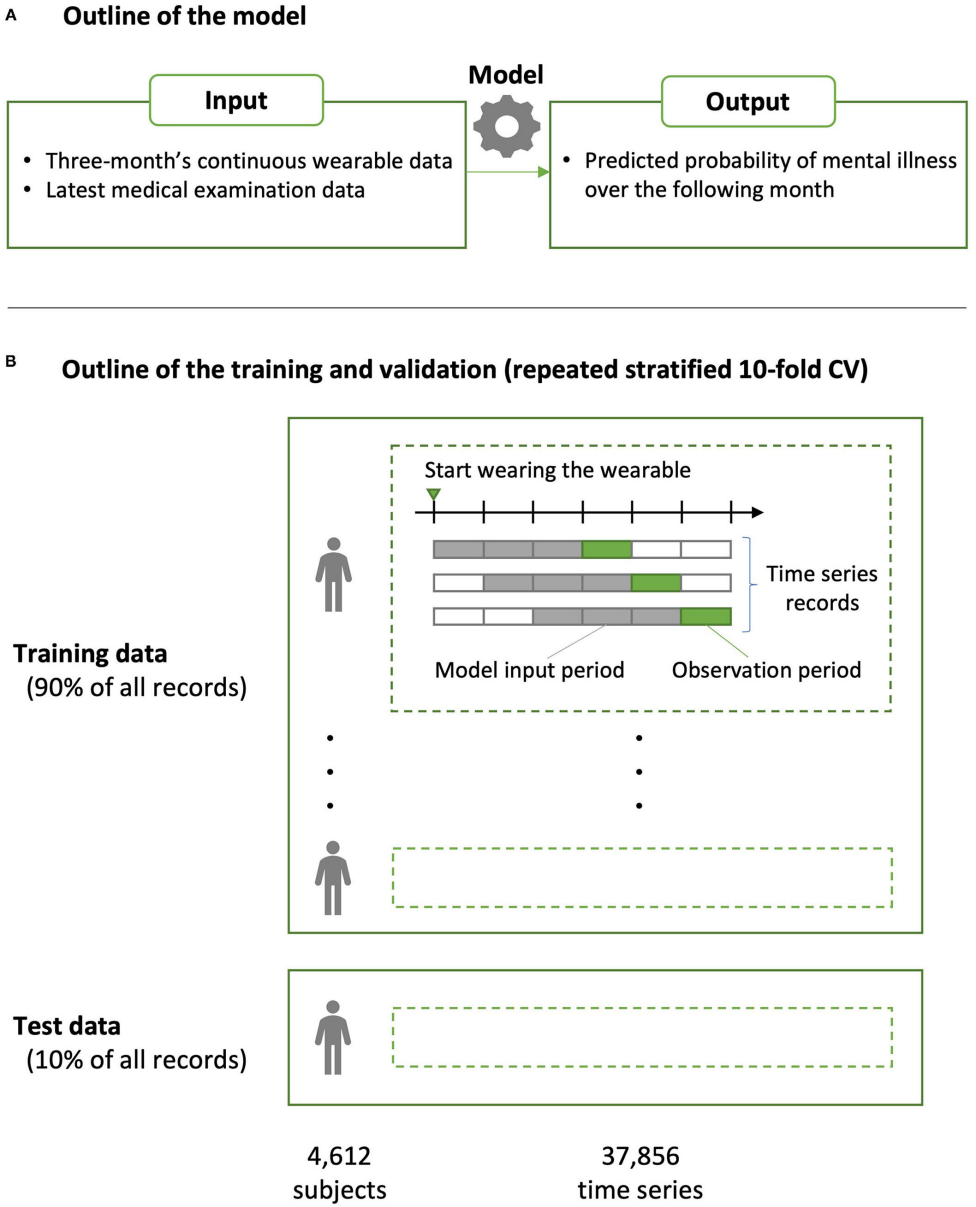
Overview of the predictive model construction and validation. **(A)** The predictive model was built using the XGBoost algorithm. The inputs to the model were 3-month's continuous wearable data and those of the medical examinations closest to (i.e., within or prior to) that period; the output was the presence or absence of mental illness over the following month, which was defined based on insurance claims data. **(B)** Predictive performance was evaluated using a repeated stratified 10-fold cross validation (CV). Because the dataset included different time series from a single person, partition division was conducted to avoid including the same person in the model training and testing data in each fold (group CV).

The XGBoost binary classification model, which is known to exhibit superior predictive performance using table data, was used as the machine learning algorithm (33). XGBoost is particularly suitable for cases where a strong correlation between features and/or a non-linear relationship between features and targets is assumed, as in this dataset. The outline of the model is shown in Figure 1. We used the “xgboost” package (v.0.81.0.1) of R analytics software (https://www.r-project.org). The loss function was a weighted logloss so that it would accommodate imbalanced data having few positive examples (34). For hyperparameters, *eta* (step size shrinkage used in update to prevent overfitting) was set at 0.05; *max_depth* (i.e., maximum depth of a tree), *min_child_weight* (i.e., minimum sum of instance weight (hessian) needed in a child), *colsample_bytree* (i.e., subsample ratio of columns when constructing each tree), and *class_weight* (i.e., weighting of positive examples in weighted logloss) were determined *via* grid search. For the other hyperparameters, the default values were used [https://xgboost.readthedocs.io/en/latest/parameter.html (accessed 2021-04-20)].

Predictive performance was evaluated *via* area-under-the-curve (AUC) using stratified 10-fold cross validation (CV). Because the dataset included different time series from a single person, partition division was conducted to avoid including the same person in the model training and testing data in each fold (group CV) (35). The validation data from each fold were merged, and the AUC for CV was evaluated for the entire training data set. This method is used for datasets having few positive examples, but it has been shown that it may underestimate performance as a classifier (36). Furthermore, the robustness of the evaluation index was ensured by conducting CV 10 times by changing the cutting method of the fold, and the average AUC was used as the predictive performance of the model constructed in this study (i.e., repeated CV).

### Statistical Analyses

The intervention range and methods of preventing mental illness onset using the predictive model were investigated by comparing the density estimation curve of the onset probability that was output by the predictive model between individuals with and without onset. A specific threshold was set for the onset probability that was output by the prediction model based on the receiver-operating-characteristic (ROC) and density estimation curves, and the statistical significance of the onset probabilities of the two groups was verified using Fisher's exact test. The subject records were created by merging the verification data of each fold in 10-fold CV.

The contribution of each feature to the outcome was confirmed using feature importance (gain), which indicates the contribution of each feature in the model-learning process. Because XGBoost is an algorithm that presumes interactions, the effect that fluctuations in each feature have on the predictive value cannot be unified and accurately shown. Therefore, we adopted a novel method of calculating the maximum value of the threshold and the fraction at which the threshold value becomes the mode value (cover) at each branch of the model built for each feature with high importance. The significance of the onset probabilities of the two groups divided by the threshold value of the total training data in this way was verified using Fisher's exact test. The *p* < 0.05 were considered statistically significant.

## Results

**Figure 2:**
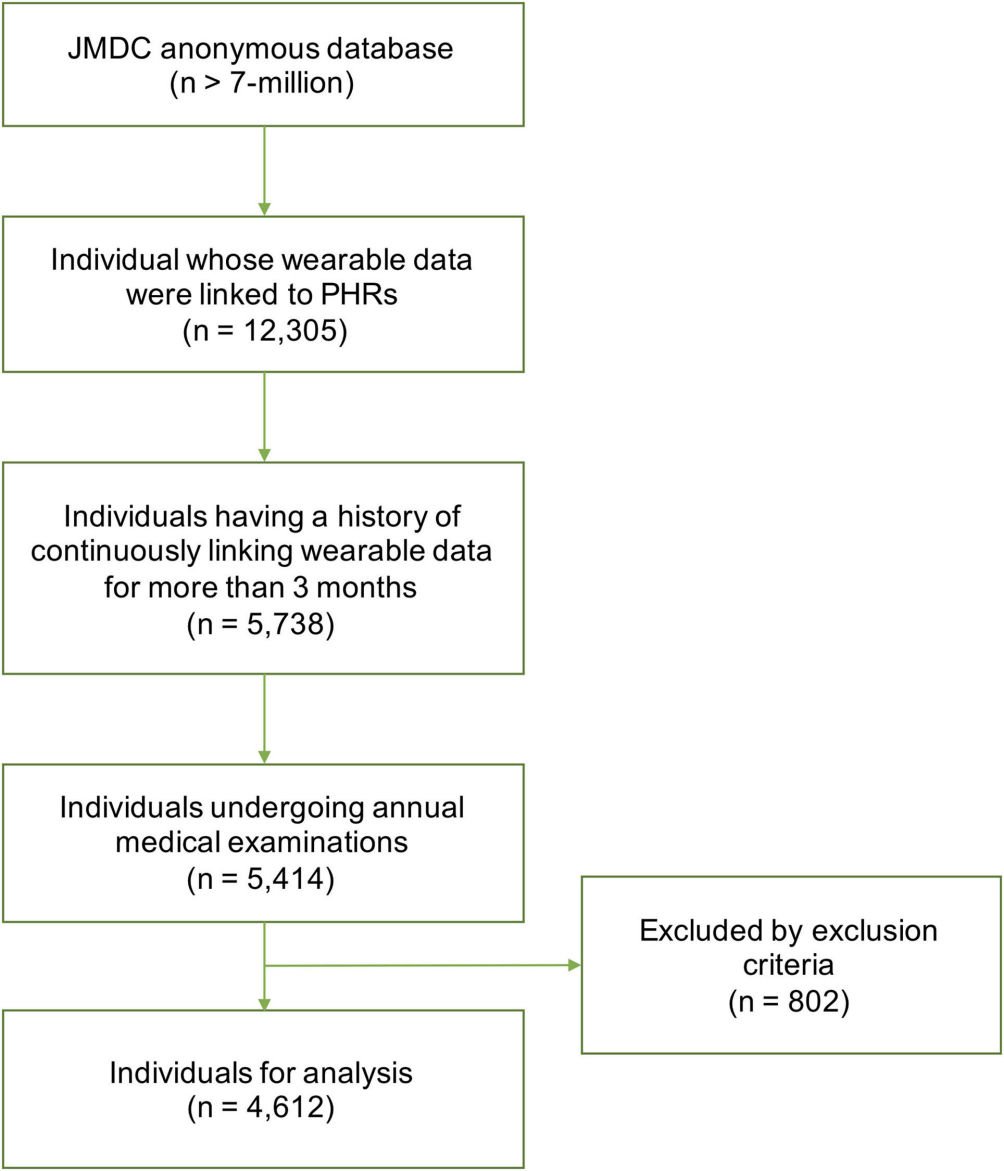
Flow diagram detailing subject inclusion. PHRs, personal health records.

The dataset comprised of 37,856 time series and 4,612 subjects (see Figure 2 for the details of subject inclusion). Table 2 shows the demographic data for the subjects. Among the subjects, 24 individuals (24 time series) exhibited mental illness onset.

**Table 2:** Demographic data of the subjects (*N* = 4,612).

Age (years), mean (*SD*)	45.9 (9.1)
Gender (male), *N* (%)	3,289 (71.3)
Body mass index (kg/m^2^), mean (*SD*)	23.2 (3.3)

*N, number of subjects; SD, standard deviation*.

**Figure 3:**
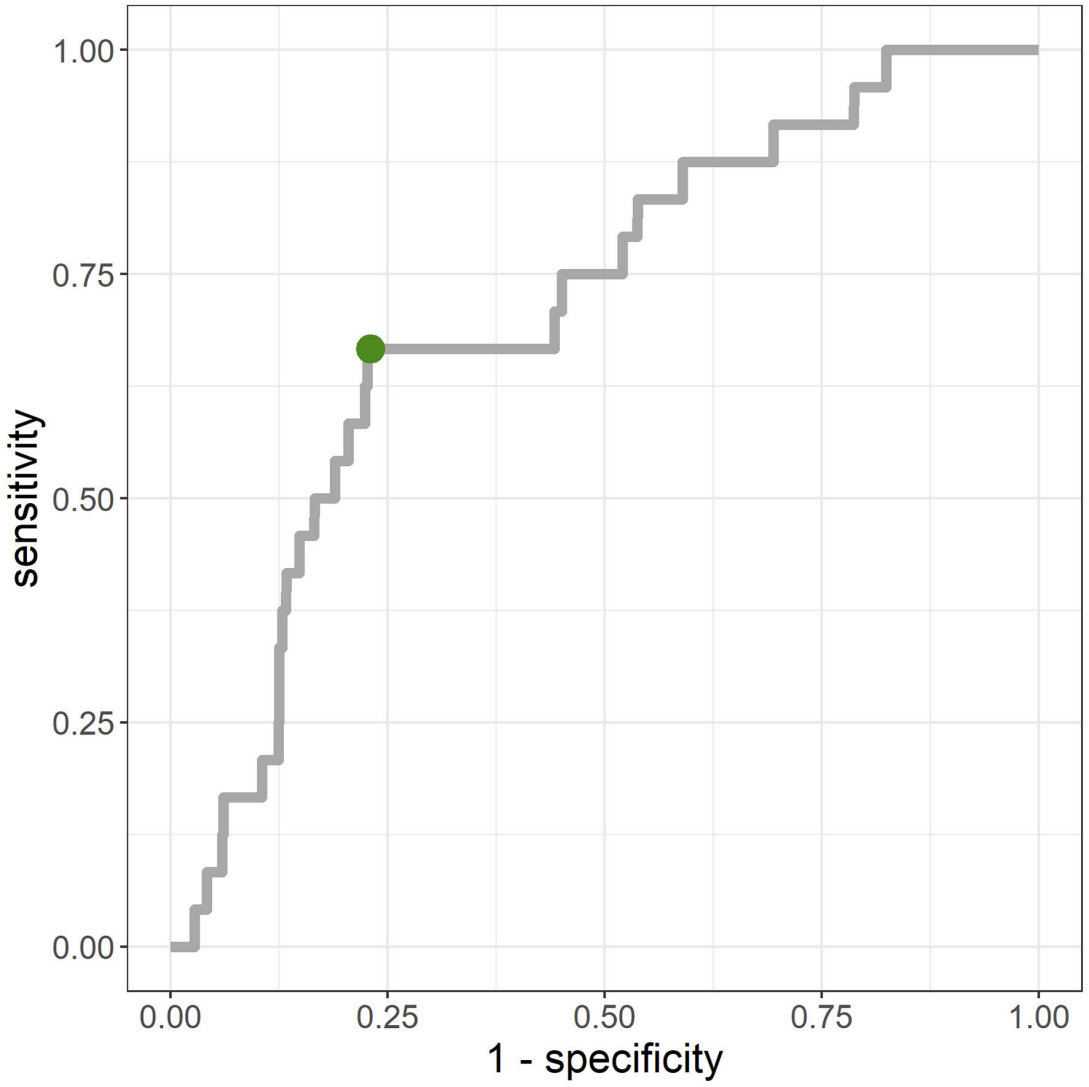
Receiver-operating-characteristic (ROC) for merged validation data created by 10-fold cross validation. Area-under-the-curve (AUC) = 0.711. The point closest to the top left (0,1) was (0.23, 0.67), and the corresponding cut-off value was 0.9%.

**Figure 4:**
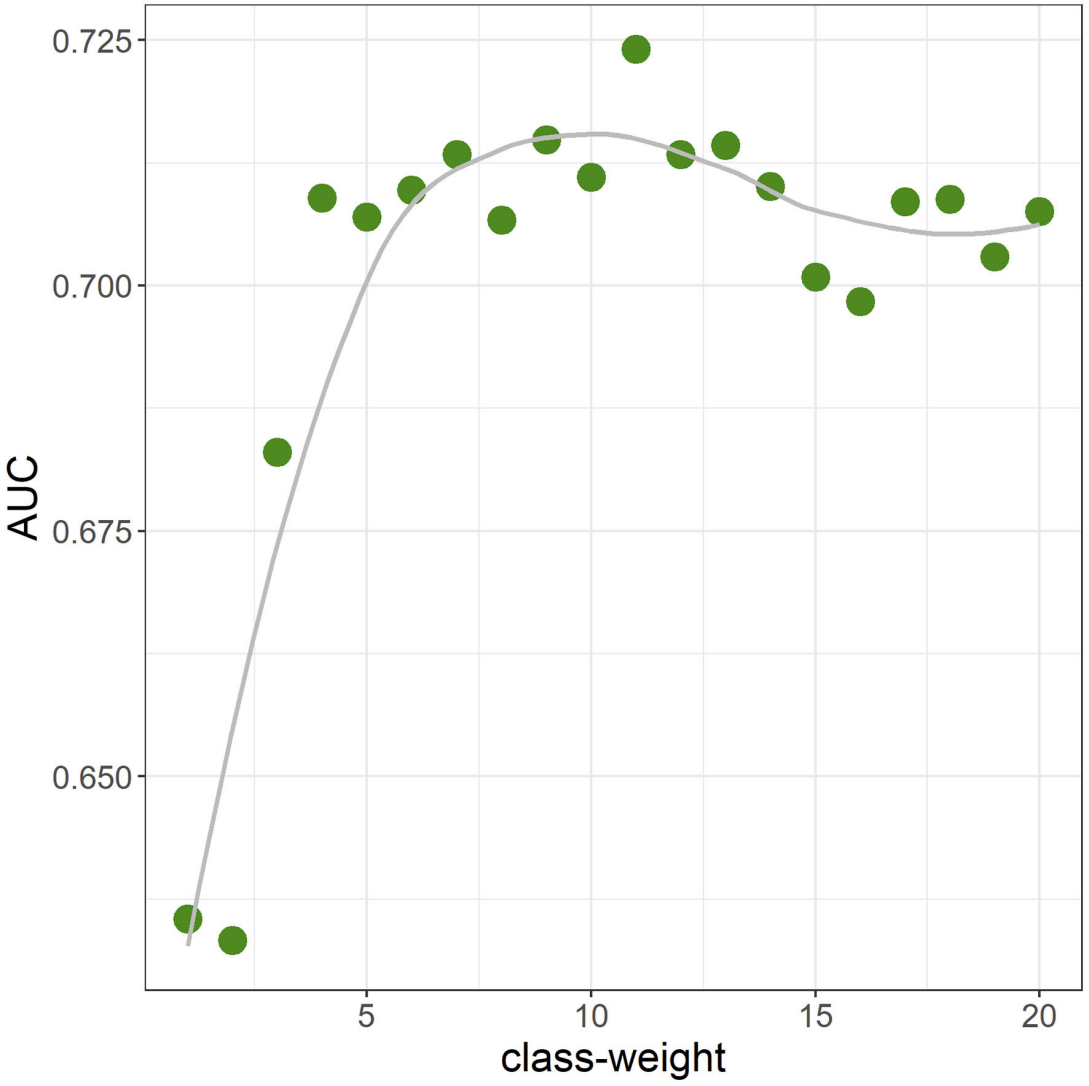
Area-under-the-curve (AUC) when the class-weight was moved from 1 to 20 in one-value increments (10-fold cross validation). Other hyperparameters were fixed at the final parameter.

The average AUC from repeated CV was 0.712 [standard deviation (*SD*) = 0.02]. Figure 3 shows the ROC curve for a given CV (AUC = 0.711). The primary hyperparameters were *max_depth* = 2, *min_child_weight* = 16, *colsample_bytree* = 0.8, *class-weight* = 10, and *nrounds* = 137. Figure 4 shows the relationship between class-weight and predictive performance; changing the loss function to a weighted logloss improved the AUC from 0.63 (*class_weight* = 1) to 0.71 (*class_weight* = 10).

**Figure 5:**
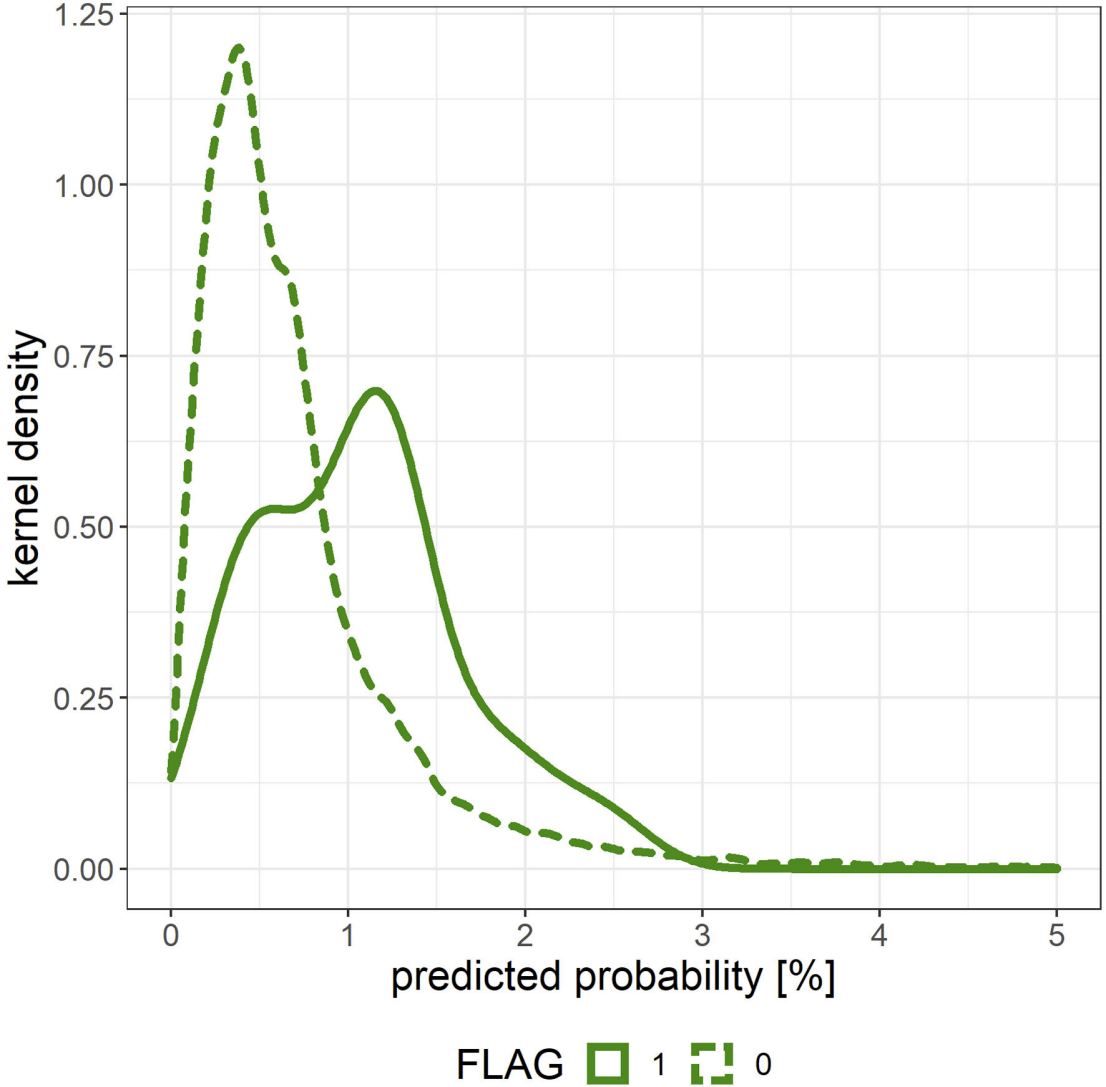
Density estimation curve of the onset probability output by the predictive model for the merged validation data created by 10-fold cross validation. The solid line (FLAG = 1) corresponds to individuals with mental illness onset, and the dashed line (FLAG = 0) corresponds to those without mental illness onset.

Figure 5 shows the density estimation curve of the onset probability output by the predictive model. When grouping this onset probability with a threshold value of 1%, the actual onset rate of the group having a predictive probability > 1% was 0.17% (*n* = 7,592, *x* = 13) and the actual onset rate of the group with predictive probability <1% was 0.04% (*n* = 30,264, *x* = 11), and there was a significant difference in the onset rates of the two groups (*p* < 0.001).

**Figure 6:**
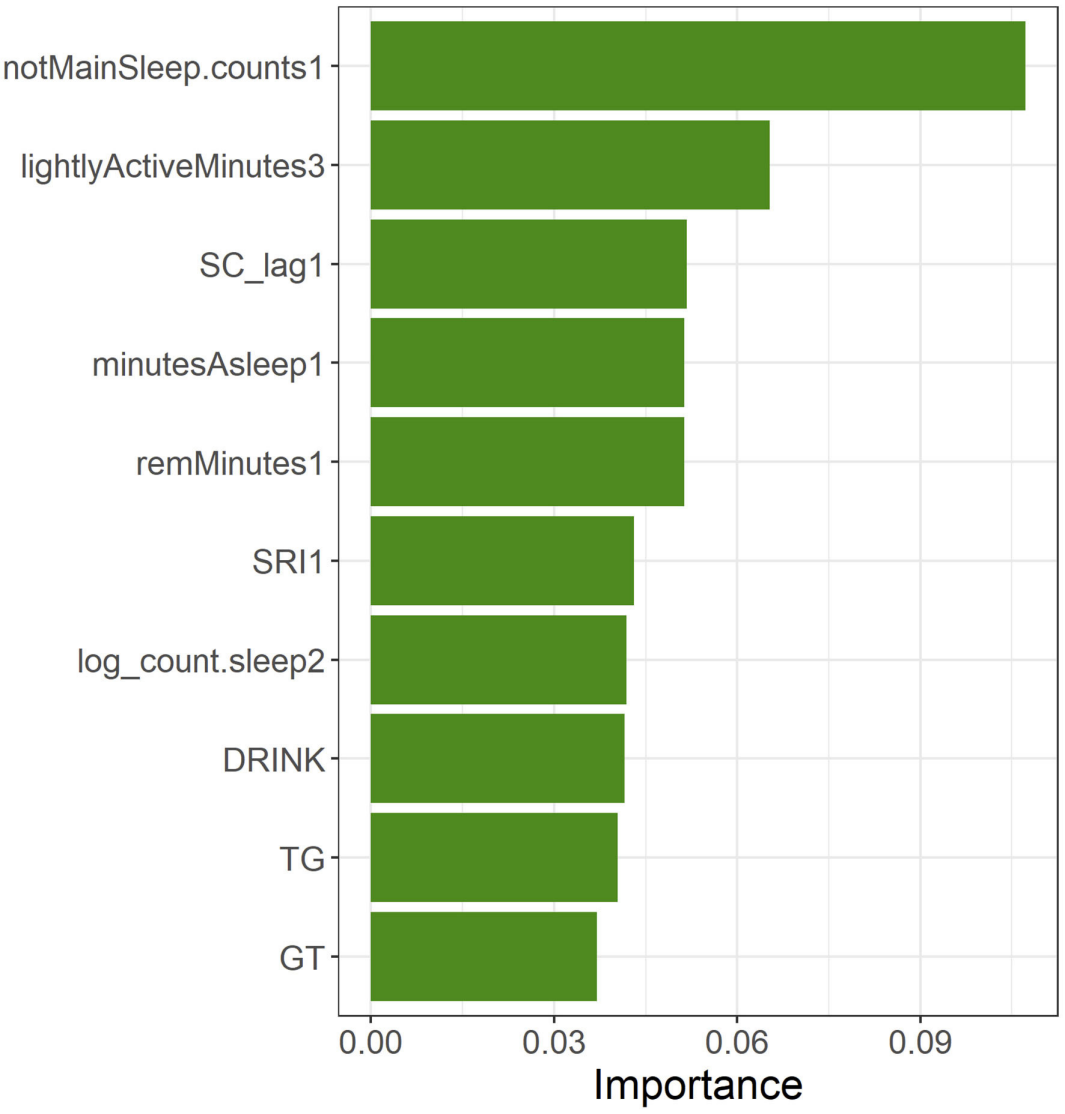
Top-10 features in feature importance (gain) of the XGBoost model built using all of the training data.

The top-10 features of the feature importance (gain) generated when the XGBoost model was built were, in order of decreasing importance, *notMainSleep.counts1, lightlyActiveMinutes3, SC_lag1, minutesAsleep1, remMinutes1, SRI1, log_count.sleep2, DRINK, TG*, and *GT* (Figure 6). When confirming the most important features of the wearable data from the perspective of the time series, sleep-related features were all in the first month, whereas the days having linked sleep data occurred during the second month, and the mild activity time occurred during the third month.

Table 3 shows the mode of the branches of the most important features and the percentage at which the threshold value became the mode value (cover) as well as the onset probabilities of the two groups. With regards to the onset probabilities in the grouping based on the threshold value in the training data, the onset probability was significantly higher in the group having a feature value higher than the threshold value than in the group having a feature value lower than the threshold for *notMainSleep.counts1, lightlyActiveMinutes3, minutesAsleep1, TG*, and *GT*. The onset probability was significantly higher in the group having a feature value lower than the threshold than in the group having a feature value higher than the threshold for *remMinutes1, SRI1*, and *log_count.sleep2*. Regarding *DRINK*, subjects with a drinking habit (threshold <2.5 for *DRINK* indicates a drinking habit; see Table 1) exhibited a higher tendency for onset probability than those without a drinking habit (*p* = 0.059). *SC_lag1* did not exhibit any significant differences in onset probability between the two groups according to mode split value (*p* = 0.278). Furthermore, *notMainSleep.counts1, lightlyActiveMinutes3, log_count.sleep2*, and *DRINK* had the same threshold for all branches (cover of 100 %), whereas *SC_lag1, SRI1*, and *TG* had a relatively low cover of around 50%.

**Table 3:** Mode values of branches in the most important features and significant differences in onset probability owing to related groups.

**Feature**	**Mode split**		**Probability**		***p-*value**
	**Value**	**Cover**	**Less than**	**More than or equal**	
notMainSleep.counts1	5	100% (62/62)	0.02% (4/20,471)	0.16% (17/10,453)	<0.001
lightlyActiveMinutes3	260	100% (20/20)	0.04% (13/30,008)	0.14% (11/7,848)	0.005
SC_lag1	105	41% (7/17)	0.06% (20/34,264)	0.11% (4/3,556)	0.278
minutesAsleep1	419	92% (23/25)	0.06% (21/36,840)	0.30% (3/1,016)	0.026
remMinutes1	75	88% (15/17)	0.15% (7/4,553)	0.03% (2/7,326)	0.032
SRI1	0.92	54% (7/13)	0.09% (22/23,637)	0.01% (2/14,219)	0.002
log_count.sleep2	30	100% (14/14)	0.09% (21/22,381)	0.02% (3/15,475)	0.006
DRINK	2.5	100% (9/9)	0.08% (21/25,439)	0.02% (2/8,946)	0.059
TG	608	54% (13/24)	0.06% (22/37,776)	2.50% (2/80)	0.001
GT	19	91% (10/11)	0.01% (1/10,431)	0.08% (23/27,425)	0.010

*“Mode split cover” is the percentage of the mode in all branches used by the applicable feature. “Probability” is the onset probability of being less than or more than the mode split value relative to the entire training data. Records missing a feature value were excluded. The “p-value” was calculated using Fisher's exact test. GT, glutamyl transferase; SRI, sleep regularity index; TG, triglyceride*.

## Discussions

### Summary of the Findings

In this study, we used machine learning to build a predictive model that used sleep and activity data acquired from Fitbit wearable devices and medical examination records to establish the criteria leading to mental illness onset. The results showed that the robust evaluation index of the constructed predictive model had an AUC that exceeded 0.7 and exhibited a moderate level of predictive performance. The relative strength of the relationship between wearable data and mental illness when compared with medical examination data was shown from the feature importance obtained. The top-ranking features of the constructed model included wearable data, specifically those of sleep-related indices. Analysis of the results of the model built with machine learning suggested that sleep abnormalities, especially the destabilization of sleep rhythms, are associated with an increased illness onset probability and that sleep disturbances may be a predictor of mental illness onset. Furthermore, activity-related indices and medical examination data relating to alcohol consumption were included in the topmost features, and these were also suggested to be factors. Early intervention aimed at sleep stabilization has been shown to be potentially effective in the prevention of mental illness.

### Possible Interpretations and Implications of the Findings

The model developed in this study had an AUC > 0.7 and was thought to have achieved a moderate level of accuracy. A predictive model was built using data acquired from a consumer-grade wearable device (i.e., Fitbit). A disadvantage of Fitbit is that its data processing algorithm has not been completely disclosed, and its details are unknown. However, the fact that a good-accuracy model was built using the machine learning XGBoost method shows that actual societal applications can be expected (37). The accuracy of consumer-grade wearable devices has continually increased, and many validation studies have discussed methods for evaluating and utilizing the quality of such devices (38–51). Recent systematic reviews relating to the measurement accuracy of sleep and physical activity by Fitbit have shown that, although there are still areas for improvement, it is fundamentally accurate (52–55). The results of this study provide evidence that precision preventive behavioral medicine (56) can be achieved by longitudinally acquiring personal health-related data using wearable devices.

The dataset used in this study was unbalanced with very few positive examples, and the prediction performance could be greatly improved by using weighted logloss as the loss function. Top-ranked features in terms of feature importance included *SRI* and *SC_lag*, and it is thought that the generation of features that reflect sleep rhythms from acquired wearable data will contribute to improved accuracy. Meanwhile, because there are many negative examples despite the high AUC, as shown at the point closest to the top left on the ROC curve in Figure 3, interventions need to be conducted on ~23% of all data-linked individuals to cover 67% of those having mental illness onset, owing to the percentage of negative cases ≥ 99%. As shown in Figure 5, the predicted probability of the model is at most a few percent per month, and even with a high threshold, many of the cases can be false positives. Although false positive cases are “healthy” in the sense that psychiatrists do not administer hypnotic, anxiolytic, or antidepressant drugs in the following month, the risk of mental health problems may be relatively high. Therefore, soft interventions based on the high rate of false positives are required in this way, and specific candidates for this include advice without concerns about side effects relating to sleep, eating habits, or exercise.

The top-seven features contributing to the prediction of onset of mental illness included information acquired by wearable devices, indicating the importance of daily life monitoring of health metrics. In particular, six out of the seven features are related to sleep and, as mentioned in many previous studies (57–61), these results suggest the possibility of a close association between sleep abnormality and mental illness onset. Because activity-related features were also ranked second in importance, and three items acquired from the medical examination data were also included in the top-10 items, physical activity and medical examination data were confirmed to contribute to the diagnosis of mental illness onset to a degree.

In this study, we not only applied machine learning to data in order to build a model having high predictive accuracy, but we also confirmed the directionality of the associations between features and illness onset probability by investigating the detailed data using the model (Table 3). The manner in which the relationship between each feature and the outcome was determined was inferred by the mode values of the branches, the percentage at which the threshold value became the mode value (cover), and the onset probabilities of two groups with a grouping based on those values. Features having a cover of over 80% where the onset probability significantly differed between groups (*p* < 0.05) (*notMainSleep.counts1, lightlyActiveMinutes3, minutesAsleep1, TG, GT, remMinutes1, SRI1*, and *log_count.sleep2*) had a monotonic relationship with the outcome, suggesting a strong correlation by them without the premise of interaction. There were no significant differences in the onset probability between groups with regard to *DRINK* (*p* = 0.059), but the cover was 100%, suggesting that interactions may strengthen its effect on the outcome. The feature importance of *SC_lag1* was ranked third, but no significant differences in onset probability were seen between groups (*p* = 0.278), and the cover was relatively low at 41%. Hence, it is suggested that the optimal threshold value changed significantly depending on whether the effect on outcome was quadratic functional (high risk even if the value was too large or too small) or by interactions. When confirming branches where that of *SC_lag1* was a mode split value (105), it can be seen that the *SC_lag1* branch was configured after (*log_count.sleep2* ≥ 30) = TRUE for all cases. When grouping was conducted based on whether *SC_lag1* was above or below the mode split value for the records with *log_count.sleep2* ≥ 30 in the training data, the onset probability of the “less than” group was 0% (0 / 14,367), and the onset probability of the “more than” group was 0.27% (3 / 1,108), with significant differences in the onset probability between groups (*p* < 0.001).

Among the sleep-related features, the most important, *notMainSleep.counts1*, was evaluated as high risk when *notMainSleep* exceeded five occurrences in a month. Increases in *notMainSleep* suggested increases in short-term sleep or polyphasic sleep, such as napping, and it is speculated that the decreased quality of *MainSleep* (i.e., sleep disturbances) is a background factor in mental illness onset. These results are consistent with the results of a previous study in which the number of naps was included in the top-ranking features used to predict the mental health status of students (18). Increases in *SC_lag1* were evaluated as high risk, and this is consistent with the results of previous studies that showed that increased social jet lag increased various health risks (24, 25). However, it should be noted that the relationship of *SC_lag1* may change according to the interactions with other features. Increases in *minutesAsleep1* were evaluated to be at high risk. The threshold value was 7 h, and these results may be counterintuitive. However, the long period regardless of sleep quality, represented by *deepMinutes*, may reflect the loss of the sleep–wake rhythm and longer periods spent lying down or increased physiological need for long periods of sleep caused by psychosomatic burdens, thereby increasing the illness onset risk. A *remMinutes1* value less than ~75 min was evaluated as high risk. A previous study using a polysomnography showed that shortened REM sleep latency was associated with depression (62), whereas the disruption and shortening of REM sleep was associated with poor physical and mental health (63–67). Some Fitbit models have been reported to have favorable REM sleep estimation accuracies (46), but care should be taken with regards to the sleep-stage estimation accuracy from consumer-grade wearable devices. Smaller *SRI1* was evaluated as high risk, and this is consistent with previous studies that stated that irregular daily sleep times led to poor mental and physical states (60, 68, 69). Unlike conventional evaluative indices, which focus only on the main sleep phase, SRI is attracting attention as an index that can evaluate the sleep timing over a 24-h period (26, 27), which is consistent with the findings related to *notMainSleep.counts*. Overall, the results suggest that the mental illness onset probability increases when the sleep–wake rhythm is disrupted, rest-activity balance is lost, and REM sleep decreases.

With regards to activity-related features, it was confirmed that *lightlyActiveMinutes3*, which was the second-highest in terms of feature importance, was evaluated as high risk when exceeding 260 min. This threshold value was in the top 20% of the overall record. Mental illness and depressive symptoms have been associated with low activity and slow movement (14–16, 70), and it is thought that there is a possibility of increased light activity intensity in the form of decreased physical activity immediately before illness onset, alongside disruptions in sleep rhythms and decreased balance.

The 8th−10th-ranked features in terms of feature importance were medical examination data, which included drinking habits (*DRINK*), triglycerides (*TG*), and γ-GTP (*GT*). All features were related to dietary lifestyle, including alcohol consumption. Drinking habits and high values indicating liver dysfunction were evaluated as high risk, and the results showed a link between dietary lifestyle and mental illness. Meanwhile, the importance of each of these features was lower than that of the wearable device-related features, and the results once again suggested the importance of wearable device data for the predictive detection of mental illness.

Focusing on temporal (monthly) information of wearable device-related features in terms of feature importance, the highly ranked sleep-related features were those from the first month, whereas the number of days with linked sleep data included features from the second month in the top rankings. This suggests the background of sleep habit disturbances first occurring prior to mental illness onset, which then lead to behavioral changes (e.g., taking off the Fitbit when going to sleep). It is interesting that the information gleaned prior to the month of the psychiatric consultation was important. The existence of a critical slowing down is known to be an early warning signal for a phase transition within complex dynamical systems (71). Moreover, reports have indicated that there are larger mood fluctuations immediately prior to the onset or termination of depression (72), as well as increased circadian rhythm instability in animal models prior to the establishment of alcohol dependence (73). From the results obtained regarding the manifestation of sleep rhythm instability, we can conclude that it provides an early warning for mental illness onset. Therefore, interventions that promote sleep improvement, in which abnormalities appear at an early stage, are thought to be effective.

Finally, although this study constructed a generalized model that predicts mental illness onset, individual differences can be understandably high. Individual differences may arise from complex interactions among biological/genetic, psychological/behavioral, and social/environmental factors. Therefore, utilizing a longitudinal personal time-series model with multiple, continuous, and objective data and records for one person should be more promising to achieve precision psychiatry (9) as well as precision preventive behavioral medicine (56). Machine learning and AI approaches should be very powerful tools for this purpose.

### Limitations

The results in this study are from a dataset having only a small number of people with mental illness (24). The feature importance shown in this study also cannot be said to be sufficiently universal. It is possible that features unique to these 24 people were captured, and that other important features were overlooked. The interactions between the features in this study may not have been sufficiently trained with just 24 positive examples, and it may not have fully captured these aspects. In fact, *max_depth* = 2 was used for hyperparameter selection by grid search, and the predictive model that was built in this study does not incorporate complicated interactions. Efforts must be made to continuously expand the dataset and to re-verify it.

The mental illness onset defined within this study's scope can be captured from health insurance claims. However, many mentally ill people do not go to the hospital, even with obvious symptoms such as insomnia and depression. Nevertheless, the threshold for psychiatric consultations is particularly high in Japan compared with other countries. Hence, using variables of “administration of hypnotics, anxiolytics, or antidepressants” and “psychiatric visits” in this study is thought to have only captured in relatively severe cases. Clinically, onset is synonymous with confirmed diagnosis. Thus, for this study, the insurance paradigm was acceptable. Mental illness generally shows high variance in symptoms as well as underlying causes. As most of the positive cases in this study are thought to be people with depression and anxiety symptoms, the cases can be used to construct disease-specific predictive models in future studies.

This study used values measured by a specific device (i.e., Fitbit); the measurement items may not provide sufficient accuracy or may have proprietary/unclear features. This would not be a problem if we were to restrict our scope to Fitbit wearers. However, when comparing these results to those obtained using other wearable devices, appliance differences must be taken into account. Although there are limits to the generalizability of the results in this study, they are compelling in that they demonstrate the possibility of predicting illness onset using wearable data.

The period of the wearable data analyzed had to be restricted to three continuous months to secure a reasonable amount of data. The closest annual medical examination data to the mental illness onset was used in the analyses, which means that the timing of the medical examination was not the same as that of the wearable data. However, medical examination data generally do not change abruptly in such a short period (~several months), which should support the validity of our study design.

Biases associated with wearable data linkages should also be noted. This was an observational study, and it is always an individual's choice to attach and use wearable devices for any reason. Therefore, there is the possibility that a person who feels physically and mentally fulfilled will continue to wear the device and link multiple time-series records. Conversely, those who feel mentally offset may discontinue wearing the device. Hence, the onset probability predicted by the model is likely lower than that actually found in society as a whole. Nevertheless, there is some value in considering the utilization of a model that focuses on the magnitude of predictive probability and conducting preventive interventions based on a given threshold value.

## Conclusions

This study demonstrated the possibility of developing a machine learning model that predicts mental illness onset using wearable data collection items and extant medical examination data. The feature importance in the predictive model developed in this study suggests that metrics such as sleep and activity cycles may be more useful in predicting the onset of mental illness than blood-test data. Sleep disturbances were detected as symptoms 3 months prior to onset, and early stage intervention that focused on improving sleep showed the potential for effective prevention.

## Data Availability Statement

The datasets presented in this article are not readily available because the data were used under license for the current study. JMDC makes the database widely available, on a fee-paying basis, for use in surveys, research, and commercial purposes. Requests to access the datasets should be directed to JMDC's website (https://www.jmdc.co.jp/en/bigdata/).

## Ethics Statement

The studies involving human participants were reviewed and approved by Ethics Committee of the Research Institute of Healthcare Data Science. The Ethics Committee waived the requirement of written informed consent for participation.

## Author Contributions

TS and AK were involved in the study conception and analytical design. TS extracted the data and performed the data analysis. TS, HS, and AK contributed to data interpretation. TS and AK wrote the first draft and all authors provided input on and approved the final manuscript. All authors contributed to the article and approved the submitted version.

## Funding

AK was supported by The University of Tokyo Excellent Young Researcher project and JST PRESTO Grant Number JPMJPR19J3, Japan.

## Conflict of Interest

TS and HS were employees of JMDC Inc. The remaining author declares that the research was conducted in the absence of any commercial or financial relationships that could be construed as a potential conflict of interest.

## Publisher's Note

All claims expressed in this article are solely those of the authors and do not necessarily represent those of their affiliated organizations, or those of the publisher, the editors and the reviewers. Any product that may be evaluated in this article, or claim that may be made by its manufacturer, is not guaranteed or endorsed by the publisher.

## References

[1] Kessler RC Angermeyer M Anthony JC De Graaf R Demyttenaere K Gasquet I. Lifetime prevalence and age-of-onset distributions of mental disorders in the World Health Organization's World Mental Health Survey Initiative. World Psychiatry. (2007) 6:168–76. 18188442 PMC2174588

[2] Steel Z Marnane C Iranpour C Chey T Jackson JW Patel V. The global prevalence of common mental disorders: a systematic review and meta-analysis 1980–2013. Int J Epidemiol. (2014) 43:476–93. 10.1093/ije/dyu038 24648481 PMC3997379

[3] Whiteford HA Degenhardt L Rehm J Baxter AJ Ferrari AJ Erskine HE. Global burden of disease attributable to mental and substance use disorders: findings from the Global Burden of Disease Study 2010. Lancet. (2013) 382:1575–86. 10.1016/S0140-6736(13)61611-6 23993280

[4] Rush AJ Trivedi M Carmody TJ Biggs MM Shores-Wilson K Ibrahim H. One-year clinical outcomes of depressed public sector outpatients: a benchmark for subsequent studies. Biol Psychiat. (2004) 56:46–53. 10.1016/j.biopsych.2004.04.005 15219472

[5] Perkins DO Gu H Boteva K Lieberman JA. Relationship between duration of untreated psychosis and outcome in first-episode schizophrenia: a critical review and meta-analysis. Am J Psychiat. (2005) 162:1785–804. 10.1176/appi.ajp.162.10.1785 16199825

[6] Zheng M Liu PX Gravina R Fortino G. An emerging wearable world: new gadgetry produces a rising tide of changes and challenges. IEEE Syst Man Cybern Mag. (2018) 4:6–14. 10.1109/MSMC.2018.2806565

[7] Mohr DC Zhang M Schueller SM. Personal sensing: understanding mental health using ubiquitous sensors and machine learning. Annu Rev Clin Psychol. (2017) 13:23–47. 10.1146/annurev-clinpsy-032816-044949 28375728 PMC6902121

[8] Dwyer DB Falkai P Koutsouleris N. Machine learning approaches for clinical psychology and psychiatry. Annu Rev Clin Psychol. (2018) 14:91–118. 10.1146/annurev-clinpsy-032816-045037 29401044

[9] Bzdok D Meyer-Lindenberg A. Machine learning for precision psychiatry: opportunities and challenges. Biol Psychiatry Cogn Neurosci Neuroimaging. (2018) 3:223–30. 10.1016/j.bpsc.2017.11.007 29486863

[10] Topol EJ. High-performance medicine: the convergence of human and artificial intelligence. Nat Med. (2019) 25:44–56. 10.1038/s41591-018-0300-7 30617339

[11] Thieme A Belgrave D Doherty G. Machine learning in mental health: a systematic review of the HCI literature to support the development of effective and implementable ML systems. ACM Trans Comput–Hum Interact. (2020) 27:1–53. 10.1145/3398069

[12] Chikersal P Doryab A Tumminia M Villalba DK Dutcher JM Liu X. Detecting depression and predicting its onset using longitudinal symptoms captured by passive sensing: a machine learning approach with robust feature selection. ACM Trans Comput–Hum Interact. (2021) 28:1–41. 10.1145/3422821

[13] Fang Y Forger DB Frank E Sen S Goldstein C. Day-to-day variability in sleep parameters and depression risk: a prospective cohort study of training physicians. NPJ Digit Med. (2021) 4:28. 10.1038/s41746-021-00400-z 33603132 PMC7892862

[14] Nakamura T Kiyono K Yoshiuchi K Nakahara R Struzik ZR Yamamoto Y. Universal scaling law in human behavioral organization. Phys Rev Lett. (2007) 99:138103. 10.1103/PhysRevLett.99.138103 17930642

[15] Sano W Nakamura T Yoshiuchi K Kitajima T Tsuchiya A Esaki Y. Enhanced persistency of resting and active periods of locomotor activity in schizophrenia. PLoS ONE. (2012) 7:e43539. 10.1371/journal.pone.0043539 22952701 PMC3429496

[16] Kim J Nakamura T Kikuchi H Yoshiuchi K Sasaki T Yamamoto Y. Covariation of depressive mood and spontaneous physical activity in major depressive disorder: toward continuous monitoring of depressive mood. IEEE J Biomed Health Inform. (2015) 19:1347–55. 10.1109/JBHI.2015.2440764 26054079

[17] Cho CH Lee T Kim MG In HP Kim L Lee HJ. Mood prediction of patients with mood disorders by machine learning using passive digital phenotypes based on the circadian rhythm: prospective observational cohort study. J Med Internet Res. (2019) 21:e11029. 10.2196/11029. Erratum in: *J Med Internet Res*. (2019) 21:e15966. 10.2196/11029 30994461 PMC6492069

[18] Sano A Taylor S McHill AW Phillips AJ Barger LK Klerman E. Identifying objective physiological markers and modifiable behaviors for self-reported stress and mental health status using wearable sensors and mobile phones: observational study. J Med Internet Res. (2018) 20:e210. 10.2196/jmir.9410 29884610 PMC6015266

[19] Setoyama D Kato TA Hashimoto R Kunugi H Hattori K Hayakawa K. Plasma metabolites predict severity of depression and suicidal ideation in psychiatric patients–a multicenter pilot analysis. PLoS ONE. (2016) 11:e0165267. 10.1371/journal.pone.0165267 27984586 PMC5161310

[20] Setoyama D Yoshino A Takamura M Okada G Iwata M Tsunetomi K. Personality classification enhances blood metabolome analysis and biotyping for major depressive disorders: two-species investigation. J Affect Disord. (2021) 279:20–30. 10.1016/j.jad.2020.09.118 33038697

[21] Nagai K Tanaka T Kodaira N Kimura S Takahashi Y Nakayama T. Data resource profile: JMDC claims databases sourced from medical institutions. J Gen Fam Med. (2020) 21:211–8. 10.1002/jgf2.367 33304714 PMC7689231

[22] Evenson KR Goto MM Furberg RD. Systematic review of the validity and reliability of consumer-wearable activity trackers. Int J Behav Nutr Phys Act. (2015) 12:159. 10.1186/s12966-015-0314-1 26684758 PMC4683756

[23] Wittmann M Dinich J Merrow M Roenneberg T. Social jetlag: misalignment of biological and social time. Chronobiol Int. (2006) 23:497–509. 10.1080/07420520500545979 16687322

[24] Baron KG Reid KJ. Circadian misalignment and health. Int Rev Psychiatry. (2014) 26:139–54. 10.3109/09540261.2014.911149 24892891 PMC4677771

[25] Roenneberg T Merrow M. The circadian clock and human health. Curr Biol. (2016) 26:R432–43. 10.1016/j.cub.2016.04.011 27218855

[26] Phillips AJK Clerx WM O'Brien CS Sano A Barger LK Picard RW. Irregular sleep/wake patterns are associated with poorer academic performance and delayed circadian and sleep/wake timing. Sci Rep. (2017) 7:3216. 10.1038/s41598-017-03171-4 28607474 PMC5468315

[27] Lunsford-Avery JR Engelhard MM Navar AM Kollins SH. Validation of the sleep regularity index in older adults and associations with cardiometabolic risk. Sci Rep. (2018) 8:14158. 10.1038/s41598-018-32402-5. Erratum in: *Sci Rep*. (2020) 10:2993 10.1038/s41598-018-32402-5 30242174 PMC6154967

[28] Horne JA Ostberg O. A self-assessment questionnaire to determine morningness-eveningness in human circadian rhythms. Int J Chronobiol. (1976) 4:97–110. 10.1037/t02254-000 1027738

[29] Roenneberg T Wirz-Justice A Merrow M. Life between clocks: daily temporal patterns of human chronotypes. J Biol Rhythms. (2003) 18:80–90. 10.1177/0748730402239679 12568247

[30] Au J Reece J. The relationship between chronotype and depressive symptoms: a meta-analysis. J Affect Disord. (2017) 218:93–104. 10.1016/j.jad.2017.04.021 28463712

[31] Taylor BJ Hasler BP. Chronotype and mental health: recent advances. Curr Psychiatry Rep. (2018) 20:59. 10.1007/s11920-018-0925-8 30039327

[32] Ojio Y Kishi A Sasaki T Togo F. Association of depressive symptoms with habitual sleep duration and sleep timing in junior high school students. Chronobiol Int. (2020) 37:877–86. 10.1080/07420528.2020.1746796 32233690

[33] Chen T Guestrin C. Xgboost: a scalable tree boosting system. In: Proceedings of the 22nd ACM SIGKDD International Conference on Knowledge Discovery and Data Mining. New York, NY: Association for Computing Machinery (2016). p. 785–94.

[34] Zadrozny B Langford J Abe N. Cost-sensitive learning by cost-proportionate example weighting. In: Proceedings of the Third IEEE International Conference on Data Mining. Melbourne, FL: IEEE (2003). p. 435–42. 10.1109/ICDM.2003.1250950

[35] Gholamiangonabadi D Kiselov N Grolinger K. Deep neural networks for human activity recognition with wearable sensors: leave-one-subject-out cross-validation for model selection. IEEE Access. (2020) 8:133982–94. 10.1109/ACCESS.2020.3010715

[36] Forman G Scholz M. Apples-to-apples in cross-validation studies: pitfalls in classifier performance measurement. ACM SIGKDD Explor Newsl. (2010) 12:49–57. 10.1145/1882471.1882479

[37] Baron KG. Feeling validated yet? A scoping review of the use of consumer-targeted wearable and mobile technology to measure and improve sleep. Sleep Med Rev. (2018) 40:151–9. 10.1016/j.smrv.2017.12.002 29395985 PMC6008167

[38] Depner CM Cheng PC Devine JK Khosla S de Zambotti M Robillard R. Wearable technologies for developing sleep and circadian biomarkers: a summary of workshop discussions. Sleep. (2020) 43:zsz254. 10.1093/sleep/zsz254 31641776 PMC7368340

[39] Svensson T Chung UI Tokuno S Nakamura M Svensson AK. A validation study of a consumer wearable sleep tracker compared to a portable EEG system in naturalistic conditions. J Psychosom Res. (2019) 126:109822. 10.1016/j.jpsychores.2019.109822 31499232

[40] Bent B Goldstein BA Kibbe WA Dunn JP. Investigating sources of inaccuracy in wearable optical heart rate sensors. NPJ Dig Med. (2020) 3:18. 10.1038/s41746-020-0226-6 32047863 PMC7010823

[41] Bliwise DL Chapple C Maislisch L Roitmann E Burtea T. A multitrait, multimethod matrix approach for a consumer-grade wrist-worn watch measuring sleep duration and continuity. Sleep. (2021) 44:zsaa141. 10.1093/sleep/zsaa141 32717070 PMC7819836

[42] de Zambotti M Cellini N Goldstone A Colrain IM Baker FC. Wearable sleep technology in clinical and research settings. Med Sci Sports Exerc. (2019) 51:1538–57. 10.1249/MSS.0000000000001947 30789439 PMC6579636

[43] Walch O Huang Y Forger D Goldstein C. Sleep stage prediction with raw acceleration and photoplethysmography heart rate data derived from a consumer wearable device. Sleep. (2019) 42:zsz180. 10.1093/sleep/zsz180 31579900 PMC6930135

[44] Wright SP Hall Brown TS Collier SR Sandberg K. How consumer physical activity monitors could transform human physiology research. Am J Physiol Regul Integr Comp Physiol. (2017) 312:R358–67. 10.1152/ajpregu.00349.2016 28052867 PMC5401997

[45] Cook JD Prairie ML Plante DT. Utility of the Fitbit Flex to evaluate sleep in major depressive disorder: a comparison against polysomnography and wrist-worn actigraphy. J Affect Disord. (2017) 217:299–305. 10.1016/j.jad.2017.04.030 28448949 PMC5509938

[46] de Zambotti M Goldstone A Claudatos S Colrain IM Baker FC. A validation study of Fitbit Charge 2™ compared with polysomnography in adults. Chronobiol Int. (2018) 35:465–76. 10.1080/07420528.2017.1413578 29235907

[47] Kahawage P Jumabhoy R Hamill K de Zambotti M Drummond SPA. Validity, potential clinical utility, and comparison of consumer and research-grade activity trackers in Insomnia Disorder I: In-lab validation against polysomnography. J Sleep Res. (2020) 29:e12931. 10.1111/jsr.12931 31626361

[48] Hamill K Jumabhoy R Kahawage P de Zambotti M Walters EM Drummond S. Validity, potential clinical utility and comparison of a consumer activity tracker and a research-grade activity tracker in insomnia disorder II: outside the laboratory. J Sleep Res. (2020) 29:e12944. 10.1111/jsr.12944 31680327

[49] Roomkham S Lovell D Cheung J Perrin D. Promises and challenges in the use of consumer-grade devices for sleep monitoring. IEEE Rev Biomed Eng. (2018) 11:53–67. 10.1109/RBME.2018.2811735 29993607

[50] Chinoy ED Cuellar JA Huwa KE Jameson JT Watson CH Bessman SC. Performance of seven consumer sleep-tracking devices compared with polysomnography. Sleep. (2021) 44:zsaa291. 10.1093/sleep/zsaa291 33378539 PMC8120339

[51] Massoomi MR Handberg EM. Increasing and evolving role of smart devices in modern medicine. Eur Cardiol. (2019) 14:181–6. 10.15420/ecr.2019.02 31933689 PMC6950456

[52] Straiton N Alharbi M Bauman A Neubeck L Gullick J Bhindi R. The validity and reliability of consumer-grade activity trackers in older, community-dwelling adults: a systematic review. Maturitas. (2018) 112:85–93. 10.1016/j.maturitas.2018.03.016 29704922

[53] Feehan LM Geldman J Sayre EC Park C Ezzat AM Yoo JY. Accuracy of Fitbit devices: systematic review and narrative syntheses of quantitative data. JMIR mHealth uHealth. (2018) 6:e10527. 10.2196/10527 30093371 PMC6107736

[54] Haghayegh S Khoshnevis S Smolensky MH Diller KR Castriotta RJ. Accuracy of wristband Fitbit models in assessing sleep: systematic review and meta-analysis. J Med Internet Res. (2019) 21:e16273. 10.2196/16273 31778122 PMC6908975

[55] Fuller D Colwell E Low J Orychock K Tobin MA Simango B. Reliability and validity of commercially available wearable devices for measuring steps, energy expenditure, and heart rate: systematic review. JMIR mHealth uHealth. (2020) 8:e18694. 10.2196/18694 32897239 PMC7509623

[56] Greenland P Hassan S. Precision preventive medicine–ready for prime time? JAMA Int Med. (2019) 179:605–6. 10.1001/jamainternmed.2019.0142 30882848

[57] Ford DE Kamerow DB. Epidemiologic study of sleep disturbances and psychiatric disorders. An opportunity for prevention? JAMA. (1989) 262:1479–84. 10.1001/jama.262.11.1479 2769898

[58] Breslau N Roth T Rosenthal L Andreski P. Sleep disturbance and psychiatric disorders: a longitudinal epidemiological study of young adults. Biol Psychiatry. (1996) 39:411–8. 10.1016/0006-3223(95)00188-3 8679786

[59] Roberts RE Shema SJ Kaplan GA Strawbridge WJ. Sleep complaints and depression in an aging cohort: a prospective perspective. Am J Psychiatry. (2000) 157:81–8. 10.1176/ajp.157.1.81 10618017

[60] Fang H Tu S Sheng J Shao A. Depression in sleep disturbance: a review on a bidirectional relationship, mechanisms and treatment. J Cell Mol Med. (2019) 23:2324–32. 10.1111/jcmm.14170 30734486 PMC6433686

[61] Freeman D Sheaves B Waite F Harvey AG Harrison PJ. Sleep disturbance and psychiatric disorders. Lancet Psychiatry. (2020) 7:628–37. 10.1016/S2215-0366(20)30136-X 32563308

[62] Arfken CL Joseph A Sandhu GR Roehrs T Douglass AB Boutros NN. The status of sleep abnormalities as a diagnostic test for major depressive disorder. J Affect Disord. (2014) 156:36–45. 10.1016/j.jad.2013.12.007 24412322

[63] Kishi A Struzik ZR Natelson BH Togo F Yamamoto Y. Dynamics of sleep stage transitions in healthy humans and patients with chronic fatigue syndrome. Am J Physiol Regul Integr Comp Physiol. (2008) 294:R1980–7. 10.1152/ajpregu.00925.2007 18417644 PMC9741833

[64] Kishi A Natelson BH Togo F Struzik ZR Rapoport DM Yamamoto Y. Sleep-stage dynamics in patients with chronic fatigue syndrome with or without fibromyalgia. Sleep. (2011) 34:1551–60. 10.5665/sleep.1396 22043126 PMC3198210

[65] Kishi A Togo F Cook DB Klapholz M Yamamoto Y Rapoport DM. The effects of exercise on dynamic sleep morphology in healthy controls and patients with chronic fatigue syndrome. Physiol Rep. (2013) 1:e00152. 10.1002/phy2.152 24400154 PMC3871467

[66] Pesonen AK Gradisar M Kuula L Short M Merikanto I Tark R. REM sleep fragmentation associated with depressive symptoms and genetic risk for depression in a community-based sample of adolescents. J Affect Disord. (2019) 245:757–63. 10.1016/j.jad.2018.11.077 30448760

[67] Leary EB Watson KT Ancoli-Israel S Redline S Yaffe K Ravelo LA. Association of rapid eye movement sleep with mortality in middle-aged and older adults. JAMA Neurol. (2020) 77:1241–51. 10.1001/jamaneurol.2020.2108 32628261 PMC7550971

[68] Brooks AT Raju S Barb JJ Kazmi N Chakravorty S Krumlauf M. Sleep regularity index in patients with alcohol dependence: daytime napping and mood disorders as correlates of interest. Int J Environ Res Public Health. (2020) 17:331. 10.3390/ijerph17010331 31947749 PMC6982308

[69] Murray JM Phillips AJK Magee M Sletten TL Gordon C Lovato N. Delayed Sleep on Melatonin (DelSoM) Study Group Sleep regularity is associated with sleep-wake and circadian timing, and mediates daytime function in Delayed Sleep-Wake Phase Disorder. Sleep Med. (2019) 58:93–101. 10.1016/j.sleep.2019.03.009 31132578

[70] Burton C McKinstry B Szentagotai Tătar A Serrano-Blanco A Pagliari C Wolters M. Activity monitoring in patients with depression: a systematic review. J Affect Disord. (2013) 145:21–8. 10.1016/j.jad.2012.07.001 22868056

[71] Scheffer M Bascompte J Brock WA Brovkin V Carpenter SR Dakos V. Early-warning signals for critical transitions. Nature. (2009) 461:53–9. 10.1038/nature08227 19727193

[72] van de Leemput IA Wichers M Cramer AO Borsboom D Tuerlinckx F Kuppens P. Critical slowing down as early warning for the onset and termination of depression. Proc Natl Acad Sci U S A. (2014) 111:87–92. 10.1073/pnas.1312114110 24324144 PMC3890822

[73] Foo JC Noori HR Yamaguchi I Vengeliene V Cosa-Linan A Nakamura T. Dynamical state transitions into addictive behavior and their early-warning signals. Proc R Soc B. (2017) 284:20170882. 10.1098/rspb.2017.0882 28768888 PMC5563804

